# Gift of a New Tropical Diseases Hospital for London

**Published:** 1919-03-15

**Authors:** 


### A REMARKABLE HOSPITAL MEETING.

### Gift of a New Tropical Diseases Hospital for London.

Interest in the annual meeting of the Seamen's Hospital Society, London, was this year so pronounced that the organisers found it necessary to change the venue from the Prince's Restaurant to His Majesty's Theatre. Even this was taxed to the full to accommodate the distinguished gathering. Rumours of forthcoming interesting announcements were in the air, and, as the following report shows, there was good foundation for the expectations raised. Before the proceedings began the orchestra of the theatre played inspiriting music of a nautical character. Admiral Sir F. Doveton Sturdee, Bt., K.C.B., K.C.M.G., Commander-in-Chief at the Nore, presided.

### Admiral Beatty's Tribute to Merchantmen.

Admiral Sir David Beatty, who it was originally intended should occupy the chair, sent the following message, which was read to the meeting :—

"It is with extreme regret that I find myself unable to be present and preside at the meeting of the Seamen's Hospital Society.

"My close association with the men of the merchant service prompts me to write this letter to beg you to express to the meeting my deep sympathy and regard for the work that is being done for wounded, sick, and injured mariners in the various establishments of the Society.

"I wish well to every branch of its work, and feel confident that the British public will ever bear in mind what they owe, not only to the men of the service to which I belong, but also to the equally gallant men of the merchant fleet." (Applause.)

New Honorary Life Governors.

The following were elected hon. life Governors in recognition of services rendered : Mrs. Angas, Mrs. Langford, Messrs. Geo Bow, W. J. Clarke, J. M. Currie, John R. B. Curtis, E. J. Drake, Major R. M. Everett, Messrs. T. P. Fisher, A. Gillespie, J. Milbourne Hay, F. W. Hudlass, O.B.E., E. D. Morton, Chas. Scholey, H. Slack, T. H. Squire, T. H. Vickery, and J. H. Wood.

Sir Perceval A. Nairne, Chairman of the Committee of Management, submitted the report for 1918, which stated that the concession by the Admiralty of a ninety-nine-years' lease of the Dreadnought Hospital placed the Society in a position of security to improve the old buildings at Greenwich and bring the establishment up to the highest state of efficiency. There had been a liberal response to the appeal for Lord Devonport's Fund for the improvement of the hospital, and long-needed alterations, it is hoped, will be commenced at no distant date.

### Mrs. Angas's Gift to the Sailor.

"One of the greatest features of the year 1918," the report continued, "has been the opening of a new branch of the Society—the Angas Convalescent Home. It is a gift to the sailor from Mrs. Angas, of Stoke Poges, in response to the appeal made by Sir Rosslyn Wemyss and Sir Henry Jackson at the last annual meeting. This is the first occasion upon which the sailor has had provided for him a home in which he can rest awhile to complete recovery after treatment in the wards of the Seamen's Hospital at Greenwich, or at the Albert Dock. It is hard to compute the value of this gift, for the sailor needs such a place of rest perhaps more than any other member of the community, as he is generally far from his home and friends when ill, and has no one to go to for that care and succour so very necessary in the trying days of convalescence. The Angas Home is at Cudham, on the North Kentish Downs. It stands on a freehold estate comprising about eighteen acres of ground, well wooded and admirably situated. The home provides accommodation for thirty patients, and was opened on July 8."

Sir Perceval Nairne also announced that beds in this home have been endowed by Mrs. Langford in memory of the late Robert Gordon; by King George's Fund for Sailors; by Sir Walter Becker; the Overseas Branch of the Navy League; Lady McIlwraith; Major R. M. Everett; and Mrs. Bury.

### The Men Who Saved the War.

Admiral Sturdee, moving that the report be adopted, agreed with what Sir David Beatty had written about the good work of the mercantile marine. Ocean highways were to Britain what railways were to Continental Powers. Let them keep that simple fact in their mind and they would know why we wanted a navy and a mercantile marine. Until the war came the men of the mercantile marine were not properly appreciated; he hoped there would be no lack of appreciation in the future. To his (Admiral Sturdee's) mind the enemy's submarine campaign was the most stupid thing the Germans ever did. It certainly failed in its purpose so far as that purpose was to destroy the *morale* of the men of the merchant service. Those men never failed. They went to sea; they were torpedoed. Some were in the water for hours, but when they reached port the first thing they did was to sign on again. (Applause.) "If the men of the mercantile marine had failed us," continued Admiral Sturdee, "Germany would have won the war. Let us not forget that there are 18,000 men of the mercantile marine at the bottom of the sea. Let us do a service to their memory by seeing that their dependants and all in the merchant service upon whom misfortune may fall shall be properly cared for." (Loud applause.)

Letter from the Hon. Sir A. Stanley.

Sir Doveton Sturdee then said he had to read to the meeting the following letter, which had been received from the Hon. Sir Arthur Stanley :

"I should have liked to be at your meeting this afternoon, but am unfortunately detained.

"I should have been glad to tell your audience, on behalf of the Joint War Committee of the Royal Red Cross Society and the Order of St. John, how glad we are to help the meeting in providing for the treatment of sailors who have contracted diseases in tropical climates in the service of their King and country.

"We are heartily glad to have it in our power to pay in this manner some small part of the vast debt that we owe to the officers and men of His Majesty's Navy and the Mercantile Service." (Applause.)

### A Noble Gift in a Noble Cause.

"I now," continued Admiral Sturdee, "have pleasure in making a very important announcement, an announcement which marks a great development in the work of the Seamen's Hospital Society. It is well known to

**Our Brave Sailors**—(*continued*).

those present that a part of the work of this Society has been the care and treatment of sailors and others suffering from tropical diseases. This branch of the Society was established at the request of the late Mr. Joseph Chamberlain (then Secretary of State for the Colonies) and Sir Patrick Manson in 1899. For twenty years patients suffering from malaria, dysentry, sleeping sickness, and suchlike diseases have been treated by experts in tropical diseases attached to the London School of Tropical Medicine.

"Now, in consequence of the largely increased number of persons suffering from tropical diseases contracted in the war, the British Red Cross Society and Order of St. John have made an offer to purchase for the Seamen's Hospital Society premises for the hospital in London, near University College, in recognition of the services rendered to the Empire by sailors in the great war. This gift will provide medical and surgical treatment for sailors and soldiers who have contracted tropical diseases while on active service, and ultimately, when this serious need has been met, it will be for the benefit of sailors.

"It has been provisionally arranged that fifty beds shall be maintained in this new branch of the Society's work. Of this number the British Red Cross Society have promised to endow thirty beds. It will be observed that there are still twenty beds to be endowed by any who sympathise with those who have contracted tropical diseases while serving their country in Mesopotamia, Egypt, Salonika, and other regions of the tropics.

"The Seamen's Hospital Society would like me to announce here that they would be pleased if the British Red Cross Society would nominate a representative to serve upon the Board, and so enable the Red Cross Society and Order of St. John to keep in touch with the Society's work. It is a noble gift in a noble cause." (Loud applause.)

### Dreadnought Hospital's Centenary.

CAPTAIN A. W. CLARKE, C.B.E. (Trinity House), in seconding the adoption of the report, drew attention to the fact that in a couple of years the Dreadnought Hospital would be celebrating its centenary. This past year had been a record in many ways, but it was possible to beat it. He was not without hope that as the result of that day's speeches some one would be inspired to come forward with open hands just as Mrs. Angas did as the result of last year's meeting. In any case, he would like to mention the fact that a bed may be endowed and named for £1,000. The fund raised by Lord Devonport for the remodelling of the Dreadnought Hospital now amounts to £150,000.

### What England Might Have Suffered.

ADMIRAL SIMS'S PRAISE.

The motion having been carried, ADMIRAL W. S. SIMS, U.S.N., moved the election of officers for the ensuing year. He sometimes doubted, he said, whether Englishmen really knew what they owe to the men who went down to the sea in ships. The American Navy had many opportunities of seeing what the merchant seamen of Britain were doing to win the war. The courage of these men was almost beyond understanding. One man who had been torpedoed four times was asked by the speaker if he was not tired of it. "No," he replied, "I am going back to sea again." **If the German submarine war had succeeded, he did not think imagination in England was sufficiently powerful to appreciate what the enemy would have done to this country. (Hear, hear.)**

### Silver Thimble Fund's Ten Thousand Pound Gift.

SIR JOHN WIMBLE, K.B.E., in seconding the motion, announced that the Silver Thimble Fund are endowing a ward in the Dreadnought Hospital by a contribution of £10,000, and therefore he urged the public to send along such gold, silver, and jewellery as they could spare. The Fund would make excellent use of it. He wished also to acknowledge the valuable assistance given to the hospital by the following: The British-American War Relief Fund, American Red Cross, Ladies' Emergency Committee of the Navy League, Queen Mary's Needlework Guild, Kensington War Hospital Supply Depot, National Egg Collection, and Vegetable Products Committee.

CAPT. ION HAMILTON BENN, C.B., supported, and the motion was carried, the proceedings closing with votes of thanks.

